# The effect of age on the association between diabetes and mortality in adult patients with COVID-19 in Mexico

**DOI:** 10.1038/s41598-021-88014-z

**Published:** 2021-04-16

**Authors:** Orison O. Woolcott, Juan P. Castilla-Bancayán

**Affiliations:** 1 Institute for Globally Distributed Open Research and Education (IGDORE), Los Angeles, CA USA; 2 Hospital Nacional Docente Madre Niño San Bartolomé, Lima, Peru; 3 Hospital Guillermo Kaelin De La Fuente, Lima, Peru

**Keywords:** Endocrinology, Diabetes, Infectious diseases, Viral infection

## Abstract

Diabetes is associated with severe COVID-19 and mortality. The aim of the present study was to determine the effect of age on the association between diabetes and mortality in patients with laboratory-confirmed COVID-19 in Mexico. This retrospective cohort study involved patients aged 20 years or older with symptoms of viral respiratory disease who were screened for SARS-CoV-2 infection across the System of Epidemiological Surveillance of Viral Respiratory Disease in Mexico from January 1 through November 4, 2020. Cox proportional-hazard regression was used to calculate the hazard ratio for 28-day mortality and its 95% confidence interval (CI). Among 757,210 patients with COVID-19 (outpatients and inpatients), 120,476 (16%) had diabetes and 80,616 died. Among 878,840 patients without COVID-19 (those who tested negative for SARS-CoV-2 infection), 88,235 (10.0%) had diabetes and 20,134 died. Among patients with COVID-19, diabetes was associated with a hazard ratio for death of 1.49 (95% CI 1.47–1.52), adjusting for age, sex, smoking habit, obesity, hypertension, immunodeficiency, and cardiovascular, pulmonary, and chronic renal disease. The strength of the association decreased with age (trend test: *P* = 0.004). For example, the adjusted hazard ratio for death was 3.12 (95% CI 2.86–3.40) for patients 20–39 years of age; in contrast, the adjusted hazard ratio of death for patients 80 years of age or older was 1.11 (95% CI 1.06–1.16). The adjusted hazard ratios were 1.66 (95% CI 1.58–1.74) in outpatients and 1.14 (95% CI 1.12–1.16) in inpatients. In hospitalized patients 80 years of age or older, no association was observed between diabetes and COVID-19-related mortality (adjusted hazard ratio: 1.03; 95% CI 0.98–1.08). Among patients without COVID-19, the adjusted hazard ratio for death was 1.78 (95% CI 1.73–1.84). In conclusion, in adult patients with COVID-19 in Mexico, the risk of death associated with diabetes decreased with age. No association between diabetes and mortality was observed among inpatients 80 years of age or older. Our findings should be verified in other populations.

## Introduction

As of March 14, 2021, over 119 million people worldwide have been infected with SARS-CoV-2, the virus that causes COVID-19. Nearly 2.65 million people have died due to COVID-19^1^.

Patients with COVID-19 who have diabetes are at increased risk of hospitalization^2,3^, admission to intensive care unit^4^, and intubation^3,5^, compared with those without diabetes. Diabetes is common among fatal cases of COVID-19^6–10^. Studies conducted during the initial months of the pandemic have reported conflicting results on the association of diabetes with COVID-19-related mortality. Some studies have shown an independent association^4,6,7,10–12^ but others have not confirmed this relationship in multivariable analysis^2,3,13–15^. These inconsistent results could be related to the relatively small number of subjects to estimate mortality risk^4,15^, the use of composite outcomes^2^, the analysis of severe COVID-19 cases or critically ill patients only^9,10,13,14^, the age of study participants and lack of stratification by age^3,6,7,10,11^, and the inclusion of unconfirmed cases^6,12^. More recent retrospective studies suggest that patients with COVID-19 who have diabetes and adequate glucose control have a lower risk of mortality compared with patients with poor glycemic control^16,17^. A growing number of studies support an independent association of diabetes with COVID-19-related mortality^18,19^.

Given the association of diabetes with severe COVID-19, specific guidelines for the treatment of patients with COVID-19 and diabetes have been proposed^20,21^. However, to date, there is a lack of age-specific guidelines for risk stratification among patients with COVID-19 who have diabetes. Although lower hazard ratios for death associated with diabetes have been reported in older subjects with COVID-19^22^, the analysis involved only critically ill patients and was restricted to three age groups. Thus, clarification on this aspect may have clinical implications for age-specific risk stratification of patients with COVID-19 who have diabetes. The aim of the present study was to determine the effect of age on the association between diabetes and mortality in patients with laboratory-confirmed COVID-19 in Mexico.

## Methods

### Study design and participants

We conducted a retrospective cohort study using data from a large sample of patients with symptoms of viral respiratory disease who were screened for SARS-CoV-2 using real-time reverse-transcriptase–polymerase-chain-reaction assay on samples obtained through oropharyngeal or nasopharyngeal swabs^23^. Patients were identified through the System of Epidemiological Surveillance of Viral Respiratory Disease in Mexico from January 1 through November 4, 2020. This surveillance system was implemented at primary, secondary or tertiary health care facilities. We included patients who were evaluated by a physician and were reported to the surveillance system from January 1 through October 7, 2020 in such a way that each patient had a 28-day follow-up unless the event (death) occurred first. We excluded patients younger than 20 years of age or coded as pregnant, and those who did not have test results for SARS-CoV-2 (Supplementary Fig. 1).

Since this study involved the analysis of publicly available de-identified data only, institutional-review-board review was not required, as outlined in the Federal Policy for the Protection of Human Subjects (detailed in 45 CFR part 46)^24^.

### Study setting

Mexico has an estimated population of 127.6 million^25^. Adults 65 years of age or older represent 6.2% of the population^26^. In Mexico, the System of Epidemiological Surveillance of Viral Respiratory Disease keeps track of suspected cases of viral respiratory disease, including COVID-19 cases, through reports from 475 Viral Respiratory Disease Monitoring Units (USMER, for its acronym in Spanish) and all health care centers (non-USMER) located nationwide. The USMER reports all suspected cases with severe respiratory symptoms but only 10% of all suspected cases with mild symptoms. The non-USMER reports all cases of severe acute respiratory infection^23^.

### Data source

Data at the individual level were obtained from the publicly available COVID-19 online dataset updated daily by the Secretary of Health of Mexico (https://www.gob.mx/salud/documentos/datos-abiertos-bases-historicas-direccion-general-de-epidemiologia). All data on demographic and pre-existing comorbidities were obtained through a standardized questionnaire^23^.

### Outcomes and definitions

The main outcome of the study was COVID-19-related death that occurred during a follow-up of 28 days from the date of patient evaluation. COVID-19-related death was defined as a deceased subject (identified using the date of death recorded in the database) who met the clinical criteria for suspected COVID-19 case and had laboratory-confirmed SARS-CoV-2 infection.

According to the guidelines from the Secretary of Health of Mexico, a suspected case of viral respiratory disease was defined as a subject who presented, in the last 10 days, cough, dyspnea, fever, or headache, and at least one of the following signs or symptoms: myalgias, arthralgias, odynophagia, chills, chest pain, rhinorrhea, polypnea, anosmia, dysgeusia, or conjunctivitis^23^. These guidelines, released on August 2020, are an update of the guidelines in which a suspected case of viral respiratory disease was defined as a subject who presented, in the last 7 days, cough, fever, or headache, accompanied with at least one of the following signs or symptoms: dyspnea, myalgias, arthralgias, odynophagia/sore throat, rhinorrhea, conjunctivitis, or chest pain^27^.

Patients who were identified at a primary health care facility and had severe respiratory symptoms were referred for advanced care at a secondary or tertiary health care facility; those who did not have severe respiratory symptoms received ambulatory treatment and were isolated at home. Patients who were identified at a secondary or tertiary facility received treatment and were isolated^23^. In the present study, a COVID-19 case was defined as a patient with suspected viral respiratory disease who had SARS-CoV-2 infection confirmed by reverse-transcriptase–polymerase-chain-reaction test. Patients who tested negative for SARS-CoV-2 infection were referred to as patients without COVID-19, regardless of epidemiological association with COVID-19. Our regression analysis included the following predictors: age, sex, smoking habit (yes/no), obesity (yes/no), hypertension (yes/no), cardiovascular disease (yes/no), chronic obstructive pulmonary disease (yes/no), asthma (yes/no), chronic kidney disease (yes/no), and immunodeficiency (yes/no). These variables were chosen among those available based on our judgment as they have been associated with the severity of COVID-19 or mortality^12,28^.

Each patient was coded as outpatient (if the patient was sent home) or inpatient (if the patient was admitted for hospitalization). This and all data used for our analyses were based on the most up-to-date information available at the time the dataset was downloaded from the website of the Secretary of Health of Mexico.

### Statistical analyses

Incidence rates of death were expressed as cases per 100,000 person-days. We used Cox proportional-hazards regression to calculate the hazard ratio for death and its 95% confidence interval (CI). We found no violation of proportional-hazards assumption. Survival was calculated using the Kaplan–Meier method. Survival time was right-censored at 28 days of follow-up. Cox regression models were adjusted for age using a five-knot restricted cubic spline fitting for age^29^. Analyses within each age group (20 to 39, 40 to 49, 50 to 59, 60 to 69, 70 to 79, and ≥ 80 years) were adjusted for age as a continuous variable. We tested for interactions between diabetes and age, diabetes and sex, and diabetes and type of patient care (outpatient or inpatient). The trends for the hazard ratios across age groups were tested using weighted linear regression. The probability weights were obtained from the inverse of the variance of the risk estimates. In the eligible population of patients, no predictor included in our regression models had more than 0.31% missing data. Thus, data were not imputed^30,31^. A complete-case analysis was performed. There were no missing data on age, sex, date of patient evaluation, date of symptoms onset, date of death, or type of patient (outpatient or inpatient).

We conducted three sensitivity analyses to assess the robustness of our findings: (1) full models with further adjustment for pneumonia, admission to intensive care unit, intubation, and time from symptoms onset to the date of patient evaluation; (2) multilevel mixed-effect survival regression models to assess the possible effect of geographical differences on our risk estimates^32^; and (3) comparison of hazard ratios from analysis restricted to patients who were evaluated and notified before and after August 1 to address the possible influence of changes to the definition of suspected viral respiratory disease^23,27^. We conducted stratified analysis according to age and sex, in outpatients and inpatients. Given the very large population, our study had statistical power to perform a robust stratified analysis in six age groups, among outpatients and inpatients. We used the log-rank test to compare unadjusted survival curves. All p values were two-sided. All analyses were performed using Stata 14 (StataCorp LP, TX).

### Ethics declarations

Since this study involved the analysis of publicly available de-identified data only, institutional review board review was not required, as outlined in the Federal Policy for the Protection of Human Subjects (detailed in 45 CFR part 46). Ethical approval or consent to participate was not required.

## Results

### Clinical characteristics

From January 1 through November 4, 2020, 2,445,709 patients of all ages with symptoms of viral respiratory disease were reported. In total, 1,650,432 patients met the study inclusion criteria. We excluded 197 patients who had implausible dates of evaluation relative to their date of death. We also excluded 14,185 patients (0.86% of all 1,650,235 eligible patients) who had missing data on diabetes, smoking habit, obesity, hypertension, cardiovascular disease, chronic obstructive pulmonary disease, asthma, chronic kidney disease, immunodeficiency, pneumonia, intubation, and admission to intensive care unit (Supplementary Table 1). The main analysis involved 757,210 adult patients with laboratory-confirmed COVID-19. We also included 878,840 adult patients who tested negative for SARS-CoV-2. In patients with COVID-19, the median age was 44 years (interquartile range [IQR], 33–56); 394,832 (52.1%) were male; 181,344 (24.0%) were hospitalized; 120,476 (15.9%) had diabetes; 139,115 (18.4%) had obesity; and 151,731 (20.0%) had hypertension (Table 1). The proportion of patients with diabetes was 10.4% among outpatients (575,866) and 33.3% among inpatients (181,344).

**Table 1:** Characteristics of study patients with COVID-19.

Characteristic	All (n = 757,210)	With diabetes (n = 120,476)	Without diabetes (n = 636,734)
Median age (IQR), years	44 (33–56)	58 (49–67)	42 (32–53)
**Age distribution, n (%)**			
20–39 years	296,881 (39.2)	9,586 (8.0)	287,295 (45.1)
40–49 years	169,645 (22.4)	22,103 (18.4)	147,542 (23.2)
50–59 years	138,707 (18.3)	34,444 (28.6)	104,263 (16.4)
60–69 years	86,446 (11.4)	30,884 (25.6)	55,562 (8.7)
70–79 years	45,352 (6.0)	17,243 (14.3)	28,109 (4.4)
≥ 80 years	20,179 (2.7)	6,216 (5.2)	13,963 (2.2)
Male sex, n (%)	394,832 (52.1)	63,431 (52.7)	331,401 (52.1)
Smoking habit, n (%)	57,451 (7.6)	9,433 (7.8)	48,018 (7.5)
Pneumonia, n (%)	140,819 (18.6)	45,895 (38.1)	94,924 (14.9)
Outpatient, n (%)	575,866 (76.1)	60,007 (49.8)	515,859 (81.0)
Hospitalized, n (%)	181,344 (24.0)	60,469 (50.2)	120,875 (19.0)
Admitted to intensive care unit, n (%)	15,501 (2.1)	5,409 (4.5)	10,092 (1.6)
Intubated, n (%)	31,921 (4.2)	11,474 (9.5)	20,447 (3.2)
Died, n (%)	80,616 (10.7)	31,389 (26.1)	49,227 (7.7)
**Pre-existing comorbidities, n (%)**			
Diabetes	120,476 (15.9)	–	–
Obesity	139,115 (18.4)	32,808 (27.2)	106,307 (16.7)
Hypertension	151,731 (20.0)	65,353 (54.3)	86,378 (13.6)
Cardiovascular disease	15,087 (2.0)	6,252 (5.2)	8,835 (1.4)
Chronic kidney disease	14,657 (1.9)	8,182 (6.8)	6,475 (1.0)
Chronic obstructive pulmonary disease	11,275 (1.5)	4,520 (3.8)	6,755 (1.1)
Asthma	19,260 (2.5)	3,251 (2.7)	16,009 (2.5)
Immunodeficiency	7,947 (1.1)	2,717 (2.3)	5,230 (0.8)
Any comorbidity including diabetes	316,917 (41.9)	–	–
Any comorbidity excluding diabetes	279,468 (36.9)	83,027 (68.9)	196,441 (30.9)
**Time variables**			
Person-days of follow-up	19,568,966	2,722,253	16,846,713
Median number of days from symptoms onset to the date of patient evaluation (IQR)	4 (2–6)	4 (2–7)	4 (2–6)
Median number of days from the date of patient evaluation to death (IQR)	6 (3–12)	6 (2–11)	7 (3–13)
Median number of days from symptoms onset to death (IQR)	12 (7–18)	11 (7–17)	12 (7–18)

In patients without COVID-19, the median age was 40 years (IQR, 30–51); 407,961 (46.4%) were male; 86,941 (9.9%) were hospitalized; 88,235 (10.0%) had diabetes; 122,550 (13.9%) had obesity; and 125,014 (14.2%) had hypertension (Supplementary Table 2). The proportion of patients with diabetes was 7.6% among outpatients (791,899) and 32.2% among inpatients (86,941).

**Table 2:** Association of diabetes with mortality among subjects with COVID-19. *

Subgroup	Number of subjects	Hazard ratio (95% CI)	
		Unadjusted	Adjusted†
All	757,210	3.74 (3.69–3.80)	1.49 (1.47–1.52)
Women	362,378	4.73 (4.62–4.84)	1.64 (1.59–1.68)
Men	394,832	3.26 (3.20–3.32)	1.41 (1.38–1.44)
**Age group‡**			
20–39	296,881	6.92 (6.40–7.48)	3.12 (2.86–3.40)
40–49	169,645	3.30 (3.16–3.45)	2.33 (2.22–2.44)
50–59	138,707	2.16 (2.10–2.23)	1.74 (1.68–1.79)
60–69	86,446	1.57 (1.53–1.61)	1.41 (1.37–1.45)
70–79	45,352	1.26 (1.23–1.30)	1.20 (1.17–1.24)
≥ 80	20,179	1.15 (1.10–1.20)	1.11 (1.06–1.16)
**Outpatients**			
All	575,866	4.90 (4.69–5.12)	1.66 (1.58–1.74)
Women	291,969	6.31 (5.87–6.78)	1.86 (1.72–2.01)
Men	283,897	4.30 (4.06–4.54)	1.55 (1.46–1.64)
**Age group‡**			
20–39	273,871	6.08 (4.79–7.70)	2.65 (2.04–3.44)
40–49	138,404	3.44 (3.02–3.92)	2.41 (2.10–2.78)
50–59	95,355	2.49 (2.28–2.72)	1.98 (1.80–2.18)
60–69	44,181	1.86 (1.71–2.02)	1.59 (1.45–1.73)
70–79	17,268	1.45 (1.32–1.60)	1.33 (1.20–1.47)
≥ 80	6,787	1.22 (1.07–1.40)	1.17 (1.01–1.35)
**Inpatients**			
All	181,344	1.42 (1.40–1.44)	1.14 (1.12–1.16)
Women	70,409	1.51 (1.47–1.55)	1.17 (1.14–1.21)
Men	110,935	1.39 (1.36–1.42)	1.12 (1.09–1.14)
**Age group‡**			
20–39	23,010	1.94 (1.79–2.11)	1.52 (1.40–1.66)
40–49	31,241	1.49 (1.43–1.56)	1.30 (1.24–1.36)
50–59	43,352	1.29 (1.25–1.33)	1.18 (1.14–1.22)
60–69	42,265	1.17 (1.14–1.20)	1.13 (1.10–1.16)
70–79	28,084	1.07 (1.04–1.11)	1.06 (1.02–1.09)
≥ 80	13,392	1.04 (0.99–1.09)	1.03 (0.98–1.08)

*Hazard ratios with 95% confidence intervals (CIs) were calculated using the Cox proportional-hazards regression.

^†^Adjusted for age, sex, smoking habit, obesity, hypertension, cardiovascular disease, chronic obstructive pulmonary disease, asthma, chronic kidney disease, and immunodeficiency. A five-knot restricted cubic spline fitting was used for age.

^‡^Hazard ratios were adjusted for age, sex, smoking habit, obesity, hypertension, cardiovascular disease, chronic obstructive pulmonary disease, asthma, chronic kidney disease, and immunodeficiency.

### Association of diabetes with mortality

As of November 4, 2020, 80,616 deaths occurred among patients with COVID-19 who were followed up for 28 days (19,568,966 person-days of observation); 31,389 (38.9%) had diabetes; 19,840 (24.6%) had obesity; and 36,764 (45.6%) had hypertension. During the same period, 20,134 deaths occurred among patients without COVID-19 (24,172,062 person-days of observation); 7,923 (39.4%) had diabetes; 3,649 (18.1%) had obesity; and 36,764 (45.6%) had hypertension (Supplementary Table 3).

In patients with COVID-19, the incidence rate of death was 1,153 cases per 100,000 person-days in those with diabetes and 292 cases per 100,000 person-days in those without diabetes. In outpatients with COVID-19, the incidence rate of death in those with and without diabetes was, respectively, 194 and 39 cases per 100,000 person-days. In hospitalized patients with COVID-19, the incidence rate of death in those with and without diabetes was, respectively, 2,553 and 1,735 cases per 100,000 person-days. In patients without COVID-19, the incidence rate of death was 345 cases per 100,000 person-days in those with diabetes and 56 cases per 100,000 person-days in those without diabetes.

**Figure 1:**
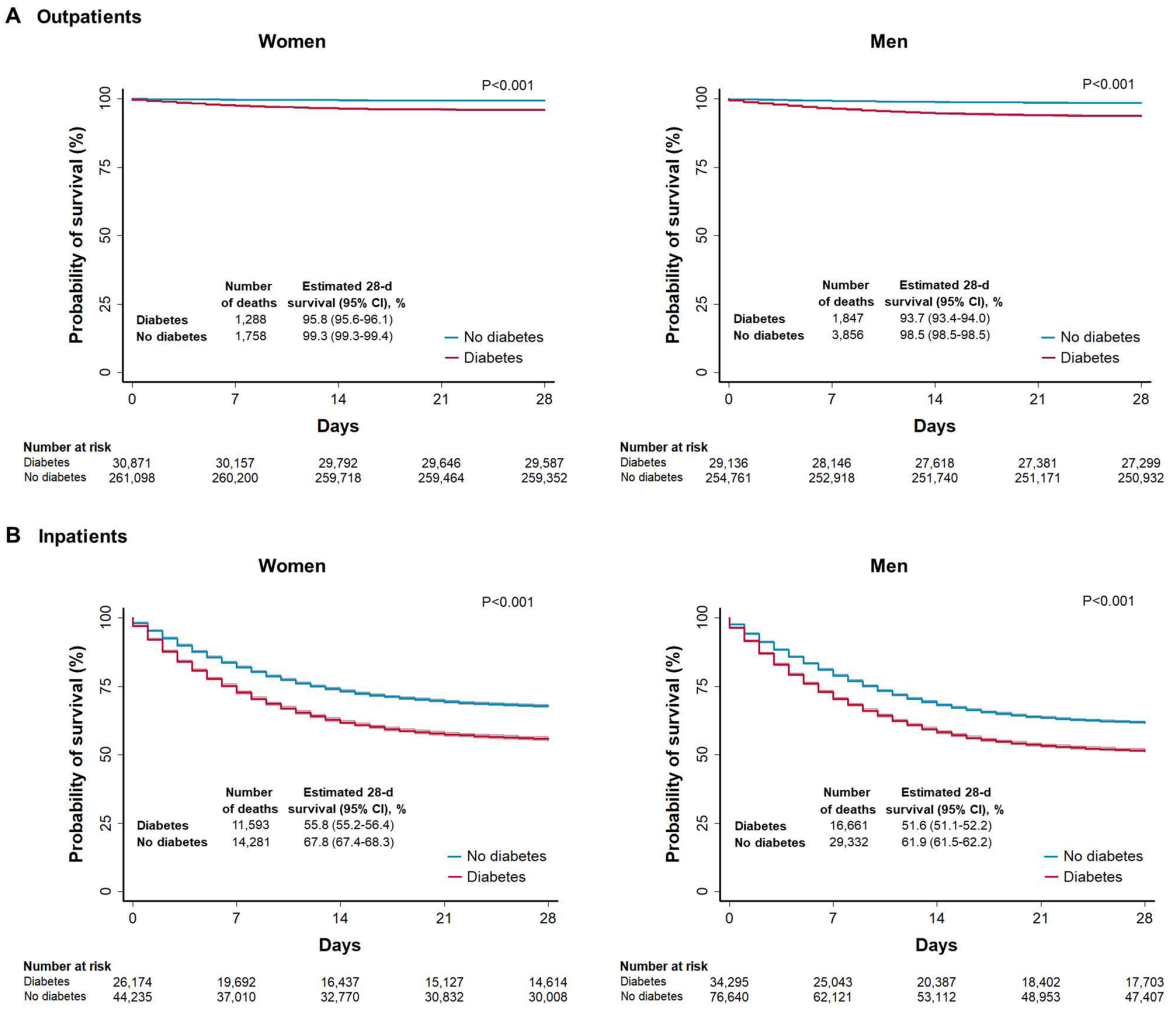
Unadjusted Kaplan–Meier survival curves among outpatients and inpatients with COVID-19 according to sex. Panels show the probability of survival according to sex among adult patients with and without diabetes who had COVID-19. Subjects were evaluated and notified from January 1 through October 7, 2020, and followed up for 28 days unless the event (death) occurred first. The solid lines represent survival probabilities and the shaded area represent the 95% confidence intervals (CIs).

Among patients with COVID-19, our adjusted Cox proportional-hazards regression analysis showed that diabetes was associated with a hazard ratio for death of 1.49 (95% CI 1.47–1.52) (Table 2). The association of diabetes with mortality was modified by age, sex, and the type of patient care (outpatient vs. inpatient) (P < 0.001 for all interactions). Men were at greater risk of death than women (hazard ratio: 1.65; 95% CI 1.63–1.68). Compared with subjects 50 to 59 years of age, those 70 to 79 years of age and those 80 years of age or older had threefold and fourfold greater risk of death, respectively (Supplementary Table 4). A slightly stronger association between diabetes and mortality was noted in women (hazard ratio: 1.64; 95% CI 1.59–1.68) than in men (hazard ratio: 1.41; 95% CI 1.38–1.44). We observed a stronger association between diabetes and mortality in outpatients (hazard ratio: 1.66; 95% CI 1.58–1.74) compared with that in hospitalized patients (hazard ratio: 1.14; 95% CI 1.12–1.16) (Table 2). Unadjusted survival curves according to sex among outpatients and inpatients with and without diabetes are shown in Fig. 1.

Among patients without COVID-19, the adjusted hazard ratio for death was 1.78 (95% CI 1.73–1.84). This association was also stronger in outpatients (hazard ratio: 1.91; 95% CI 1.68–2.18) compared with that in hospitalized patients (hazard ratio: 1.11; 95% CI 1.07–1.14) (P < 0.001 for interaction). The 28-day unadjusted survival for inpatients with diabetes who had COVID-19 was lower (53.4%) compared with that for those without COVID-19 (73.4%) (Supplementary Fig. 2).

**Figure 2:**
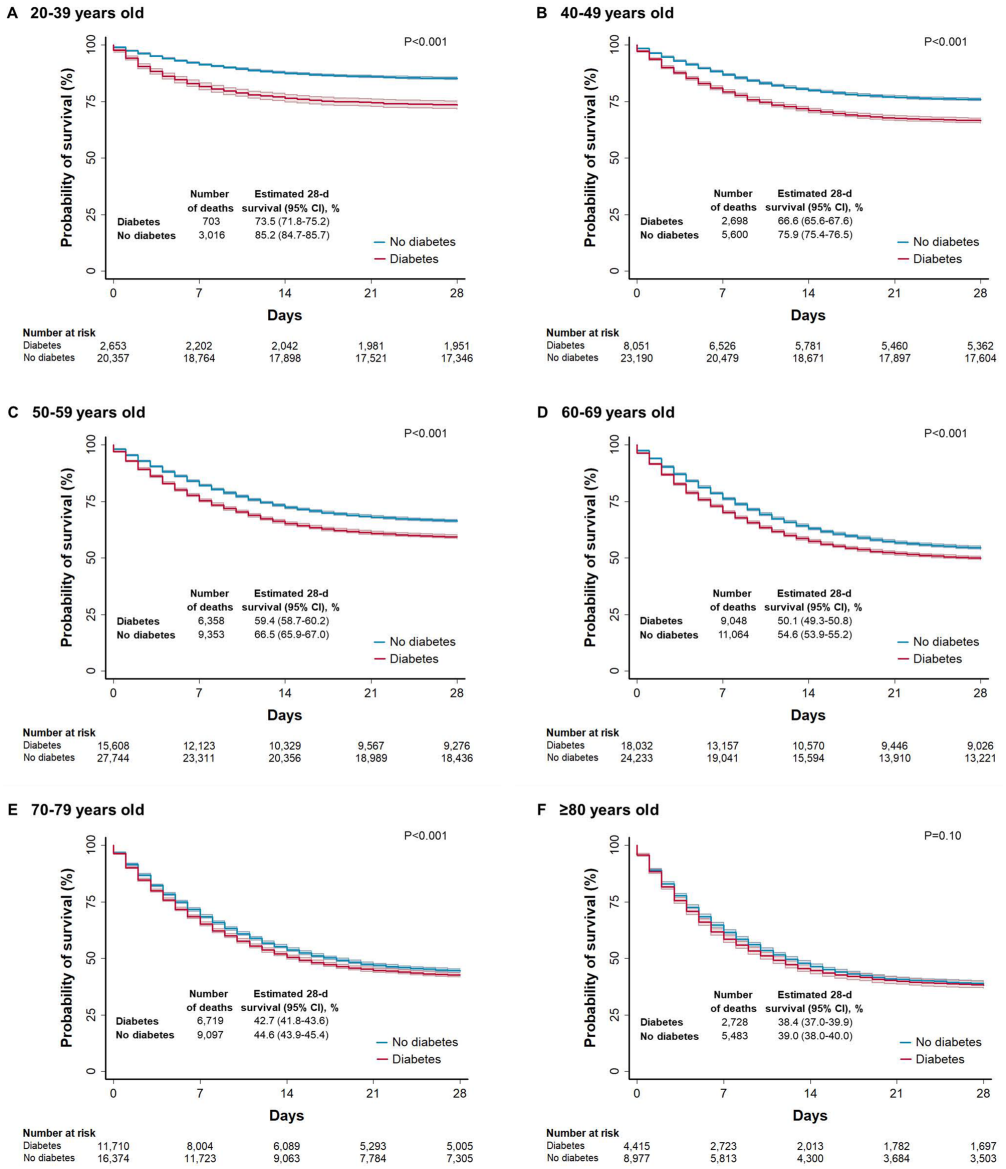
Unadjusted Kaplan–Meier survival curves according to age for inpatients with COVID-19. Panels A to F show the probability of survival stratified according to age groups among adult inpatients with and without diabetes who had COVID-19. Subjects were evaluated and notified from January 1 through October 7, 2020, and followed up for 28 days unless the event (death) occurred first. The solid lines represent survival probabilities and the shaded area represent the 95% confidence intervals (CIs).

### Sensitivity analyses

In patients with COVID-19, the association of diabetes with mortality persisted after further adjustment for pneumonia, admission to intensive care unit, intubation, and time from symptoms onset to the date of patient evaluation (Supplementary Table 5). Accounting for geographical location did not substantially affect our estimates of the risk of death for outpatients (hazard ratio: 1.62, 95% CI 1.54–1.70) or inpatients (hazard ratio: 1.13, 95% CI 1.12–1.15) with COVID-19. The updated guidelines to define suspected cases of viral respiratory disease did not have a substantial effect on the hazard ratios estimates for outpatients (before: 1.66, 95% CI 1.57–1.74; after: 1.63, 95% CI 1.42–1.87) or inpatients (before: 1.14, 95% CI 1.12–1.16; after: 1.15, 95% CI 1.10–1.19).

### Stratified analysis by sex and age groups

In stratified analysis among patients with COVID-19 (outpatients and inpatients together) according to sex and age, diabetes was associated with higher mortality in all age groups, among women and men. We observed that the risk of death associated with diabetes decreased with age. The hazard ratio for death was 3.12 (2.86–3.40) for patients 20–39 years of age; 2.33 (2.22–2.44) for patients 40–49 years of age; 1.74 (1.68–1.79) for patients 50–59 years of age; 1.41 (1.37–1.45) for patients 60–69 years of age; 1.20 (1.17–1.24) for patients 70–79 years of age; and 1.11 (1.06–1.16) for patients 80 years of age or older (Table 2) (trend test: P = 0.004). These trends were observed among outpatients (P = 0.001) and inpatients (P = 0.006) (Table 2), and among women (P = 0.006) and men (P = 0.007) (Supplementary Table 6). Among outpatients, diabetes was associated with mortality across all age groups. In contrast, we did not observe an association among inpatients 80 years of age or older (hazard ratio: 1.03; 0.98–1.08). Although the risk of death decreased with age, the incidence rate of death among patients with diabetes increased with age (Supplementary Table 7). The risk of death associated with obesity also decreased with age (P < 0.001). However, we did not observe an association among patients 80 years of age or older (hazard ratio: 1.06; 0.99–1.12). Although the risk of death associated with hypertension did not decreased linearly with age (P = 0.34), hypertension was associated with mortality across all age groups.

In hospitalized patients with COVID-19, the unadjusted survival at 28 days of follow-up for those with diabetes compared with that among those without diabetes decreased as age increased. We did not observe substantial differences in survival between patients 70 years of age or older with diabetes and those without diabetes (Fig. 2). The corresponding unadjusted survival curves in hospitalized patients without COVID-19 are shown in Supplementary Fig. 3.

## Discussion

Our analysis of a large population of adult patients with COVID-19 in Mexico shows that, overall, those with diabetes have nearly 50% greater risk of 28-day mortality compared with those without diabetes, adjusting for age, sex, smoking habit, and other pre-existing comorbidities. The strength of the association decreased with age. For example, the adjusted hazard ratio for death was 3.12 for patients 20–39 years of age; in contrast, the adjusted hazard ratio for patients 80 years of age or older was 1.11. The magnitude of the association between diabetes and mortality was stronger in outpatients than in inpatients. Among inpatients 80 years of age or older, this study did not find an association between diabetes and COVID-19-related mortality.

Lower hazard ratios for death associated with diabetes have been previously reported in older critically ill patients with COVID-19 in England^22^. However, the analysis was restricted to three age groups. A small meta-analysis involving patients with COVID-19 who had pneumonia^33^, mostly from China, showed that age affected the association between diabetes and a composite outcome of mortality, severe COVID-19, acute respiratory distress syndrome, need for intensive care, and disease progression. Our study was performed in a population with a number of deaths that was 15 times as high as the number analyzed in the former study^22^, which enabled us to conduct a more comprehensive analysis by age groups. Our findings clearly show that the risk of death associated with diabetes decreases with age, both in outpatients and inpatients with COVID-19 (Table 2).

Although diabetes was associated with COVID-19-related mortality among outpatients 80 years of age or older, no association was observed among inpatients 80 years of age or older. Although there is no clear explanation for these findings, one possibility is the effect of residual confounding. We observed a higher prevalence of pre-existing comorbidities among inpatients than among outpatients. Although our analyses were adjusted for numerous pre-existing comorbidities, the effect of unreported comorbidities may have attenuated the association of diabetes with COVID-19-related mortality among inpatients and to a lesser extent among outpatients. For example, it has been reported that neurodegenerative diseases, commonly present among older institutionalized patients^34,35^, are independently associated with mortality in patients with COVID-19^34^. The possible confounding effect of other comorbidities unaccounted in the present study on the association of diabetes with mortality in patients with COVID-19 requires further research.

We also observed that the 28-day unadjusted survival probability for inpatients with diabetes in the youngest group (20 to 39 years) was about 12 percentage points lower compared with that for inpatients without diabetes. In contrast, this difference was less than 2 percentage points in inpatients 70 years of age or older. We found that diabetes considerably increases the risk of mortality in young adults who have COVID-19 compared with that among those without diabetes, which is consistent with findings from other studies^22,36^. Although it is unclear why the association between diabetes and COVID-19-related mortality was weaker in older patients, it is possible that unreported pre-existing comorbidities, more often seen in older patients than in younger patients, may have attenuated the association of diabetes with mortality in older patients with COVID-19. It should be noted that our findings shown in Supplementary Table 4 and those from other studies^2,7,12^ clearly indicate that age is the strongest independent predictor of mortality in patients who had COVID-19. Thus, an alternative explanation is that diabetes may not further increase the risk of mortality in patients with advanced age as old patients are already at very high risk.

Few studies have examined the association of diabetes with COVID-19-related mortality in outpatients^11,12^. We observed a substantial difference in the magnitude of the association of diabetes with COVID-19-related mortality between outpatients and inpatients, suggesting this association is weaker in patients with more severe COVID-19. However, this should be interpreted in the context of the mortality rates in these groups. The difference in the incidence rate of deaths between patients with and without diabetes was five times greater in hospitalized patients than in outpatients.

Although diabetes is very common in patients with COVID-19^20,21^, in our study, the proportion of patients with diabetes among inpatients was similarly high in those with and without COVID-19 (~ 30%). Among deceased patients, the proportion of patients with diabetes was also similar in both groups (~ 40%). Diabetes was associated with mortality in patients with COVID-19, but this association was not stronger than that observed in patients without COVID-19. It should be noted, however, that the incidence rate of death among patients with diabetes and COVID-19 was 3.3 times greater than that among patients with diabetes without COVID-19, suggesting SARS-CoV-2 infection is by far the strongest predictor of mortality in this population. Among the 1.6 million study patients who were screened for SARS-CoV-2 infection, those with a positive test had 3.7 times greater risk of mortality than those with a negative test, adjusting for age, sex, smoking habit, diabetes, and other pre-existing comorbidities.

Our study has strengths and limitations. The high number of fatal cases among study patients with laboratory-confirmed COVID-19 (> 80,000 deaths) and the high number of patients who had diabetes (> 120,000) enabled us to obtain precise estimates of the association of diabetes with COVID-19-related mortality and conduct a robust stratified analysis according to age groups, among outpatients and inpatients. Limitations of this study include self-reported diabetes, unknown type of diabetes, and unknown diabetes status. Our estimates were not adjusted for ethnicity or clinical and laboratory variables since data were not available. Better blood glucose control has been associated with lower COVID-19-related mortality^16,17^. Thus, the diabetes status of the participants, unaccounted in our study, may have affected our estimates. Another limitation is that we cannot exclude the possibility that the number of deaths in patients who had COVID-19 could be underreported. Likewise, we cannot exclude the possibility that some outpatients may have required hospitalization as their symptoms worsened but they were not hospitalized due the collapse of the health care system (e.g., related to limited number of hospital beds, intensive care unit beds, and mechanical ventilators). This may also explain the low number of patients who were admitted to intensive care unit (~ 15,000) or were intubated (~ 32,000), despite the high mortality (> 80,000). Finally, since our analysis was restricted to patients who presented symptoms of viral respiratory disease and only 10% of patients with mild symptoms (at the time of the examination) were reported to the surveillance system, our findings may not be generalizable to populations with asymptomatic SARS-CoV-2 infection or mild COVID-19.

In our analysis of a large population of adult patients with laboratory-confirmed COVID-19 in Mexico, diabetes was independently associated with a 50% greater risk of 28-day mortality. The magnitude of this association was lower than those reported in previous studies in England (80 to 90% higher risk) that included laboratory-confirmed and unconfirmed cases of COVID-19^6,12^. Our data also showed that the association of diabetes with COVID-19-related mortality differed between outpatients and inpatients, and decreased with age. Among hospitalized patients, we observed a modest association in patients 70 to 79 years of age. No association was found in hospitalized patients 80 years of age or older. In the present study, the latter age group represented 11% of all COVID-19-related deaths among hospitalized patients. Data from studies in the US, UK, and Italy^12,28,37^ indicate that 43% to 59% of all COVID-19-related deaths occurred in patients 80 years of age or older. Further research is also needed to determine whether age modifies the association of diabetes with COVID-19-related mortality among patients with good and poor long-term glycemic control.

In conclusion, in adult patients with COVID-19 in Mexico, the risk of death associated with diabetes decreased with age. No association between diabetes and mortality was observed among inpatients 80 years of age or older. Our findings should be verified in other populations.

## Supplementary Information

Supplementary Information

## Data Availability

The dataset analyzed during the current study is publicly available from the Secretary of Health of Mexico: https://www.gob.mx/salud/documentos/datos-abiertos-bases-historicas-direccion-general-de-epidemiologia.
