# Supplementary material for: The effect of age on the association between diabetes and mortality in adult patients with COVID-19 in Mexico
Source: Sci Rep. 2021 Apr 16;11:8386. doi: 10.1038/s41598-021-88014-z (PMC8052362; doi:10.1038/s41598-021-88014-z)
Supplement: Supplementary file 1 — Supplementary Information [file 41598_2021_88014_MOESM1_ESM.pdf]

Supplemental Material

Supplement to: Woolcott OO, Castilla-Bancayán JP. The effect of age on the association between diabetes and mortality in adult patients with COVID-19 in Mexico

Table of Contents

Supplementary Figure 1. Flow diagram of selection of study patients. 3

Supplementary Figure 2. Comparison of unadjusted Kaplan-Meier survival curves among subjects with and without COVID-19. 4

Supplementary Figure 3. Unadjusted Kaplan-Meier survival curves according to age among inpatients without COVID-19. 5

Supplementary Table 1. Characteristics of study patients eligible for analysis. 6

Supplementary Table 2. Characteristics of study patients without COVID-19. 7

Supplementary Table 3. Characteristics of deceased subjects. 8

Supplementary Table 4. Association of age, sex, pre-existing clinical conditions, and smoking habit with mortality among subjects with COVID-19. 9

Supplementary Table 5. Sensitivity analysis for the association of diabetes with mortality among subjects with COVID-19. 10

Supplementary Table 6. Association of diabetes with mortality among subjects with COVID-19 according to age and sex. 11

Supplementary Table 7. Incidence rates of mortality among subjects with COVID-19 according to age and sex. 12

Supplementary Figure 1. Flow diagram of selection of study patients.

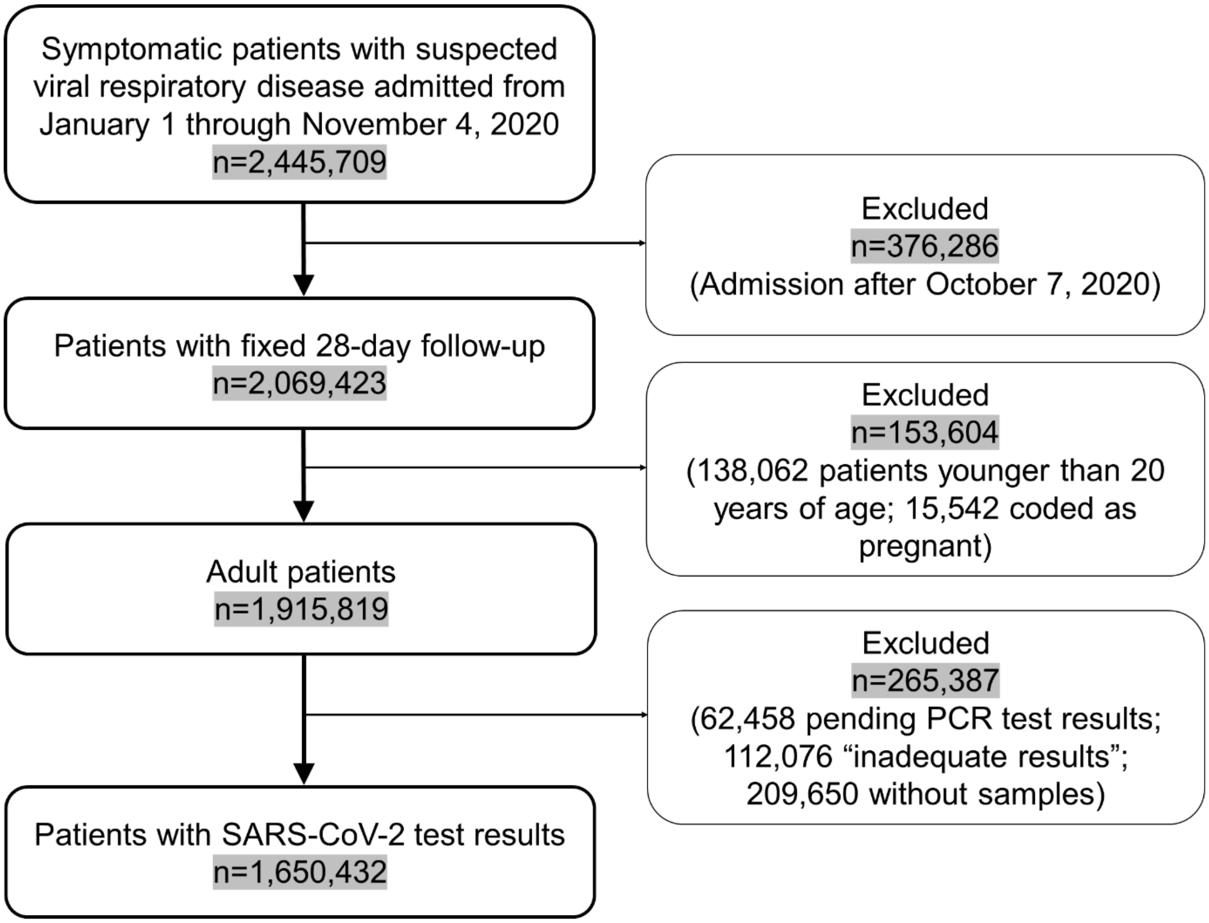

Supplementary Figure 2. Comparison of unadjusted Kaplan-Meier survival curves among subjects with and without COVID-19. Panels show the probability of survival among outpatients (A) and inpatients (B) who had diabetes with and without COVID-19. Subjects were evaluated by a physician and reported to the surveillance system from January 1 through October 7, 2020, and followed up for 28 days unless the event (death) occurred first. The solid lines represent survival probabilities and the shaded area represent the 95% confidence intervals (CIs).

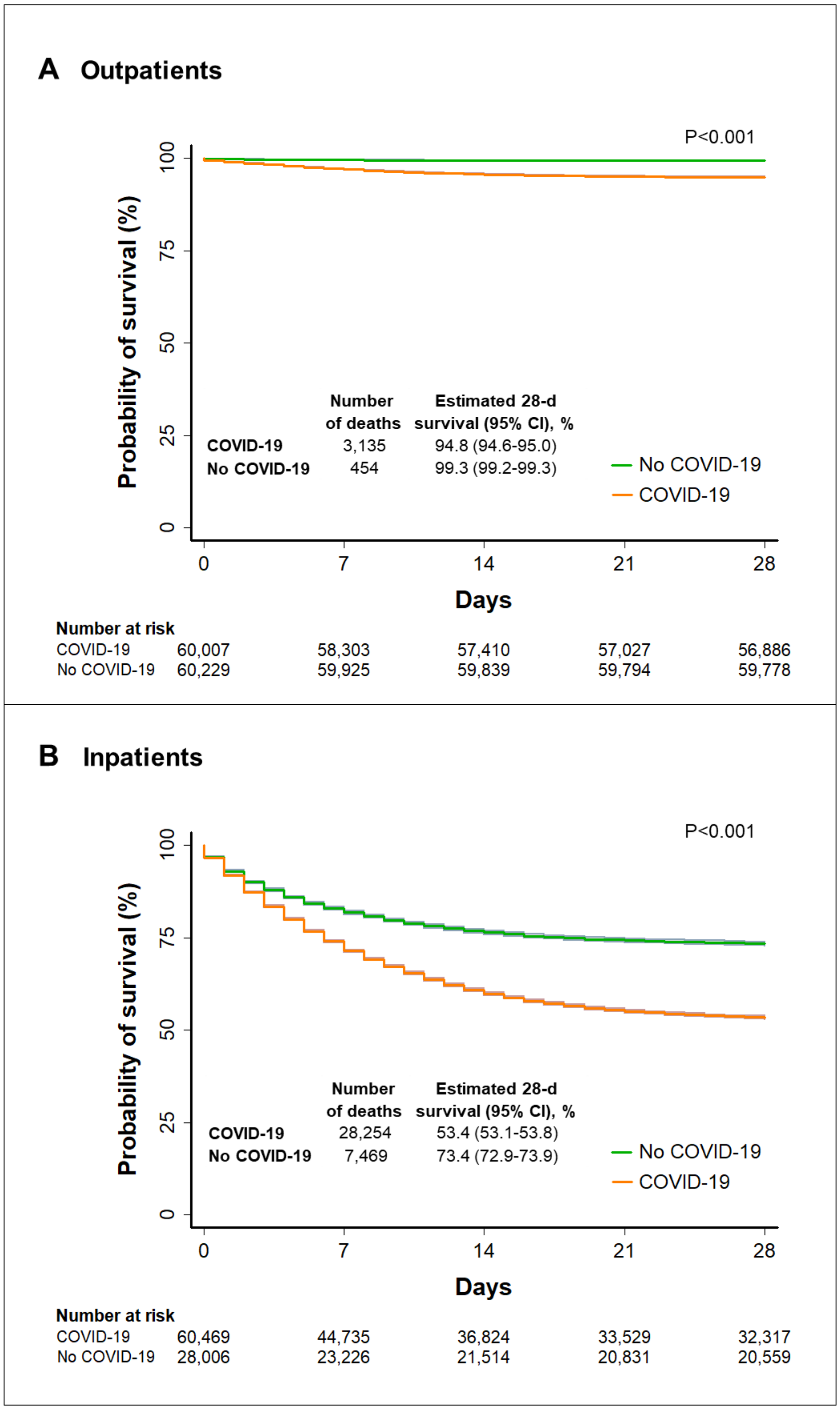

Supplementary Figure 3. Unadjusted Kaplan-Meier survival curves according to age among inpatients without COVID-19. Subjects were enrolled from January 1 through October 7, 2020, and followed up for 28 days unless the event (death) occurred first. The solid lines represent survival probabilities and the shaded area represent the 95% confidence intervals (CIs).

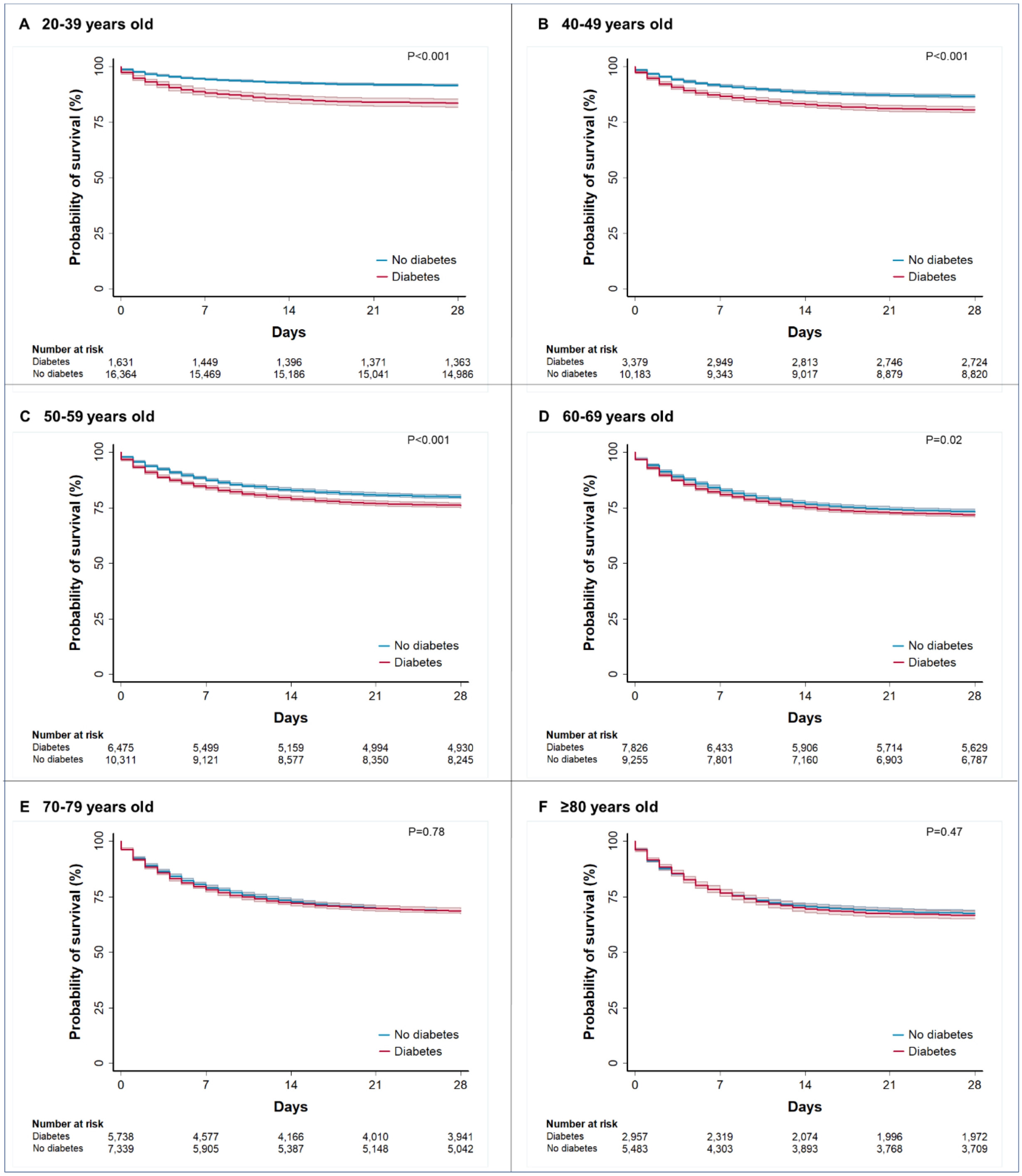

Supplementary Table 1. Characteristics of study patients eligible for analysis.

Characteristic	Included (n=1,636,050)	Excluded*		
		All (n=14,185)	With COVID-19 (n=4,461)	Without COVID-19 (n=9,724)
Median age (IQR), years	42 (32-54)	48 (35-62)	51 (39-63)	46 (33-61)
Age distribution, n (%)				
20-39 years	727,106 (44.4)	4,844 (34.2)	1,183 (26.5)	3,661 (37.7)
40-49 years	365,495 (22.3)	2,738 (19.3)	886 (19.9)	1,852 (19.1)
50-59 years	275,110 (16.8)	2,601 (18.3)	975 (21.9)	1,626 (16.7)
60-69 years	154,676 (9.5)	1,857 (13.1)	737 (16.5)	1,120 (11.5)
70-79 years	77,195 (4.7)	1,200 (8.5)	454 (10.2)	746 (7.7)
≥80 years	36,468 (2.2)	945 (6.7)	226 (5.1)	719 (7.4)
Male sex, n (%)	802,793 (49.1)	7,269 (51.2)	2,592 (58.1)	4,677 (48.1)
SARS-CoV-2 infection, n (%)	757,210 (46.3)	4,461 (31.5)	4,461 (100)	--
Outpatient, n (%)	1,367,765 (83.6)	7,602 (53.6)	2,475 (55.5)	5,127 (52.7)
Hospitalized, n (%)	268,285 (16.4)	6,583 (46.4)	1,986 (44.5)	4,597 (47.3)
Died, n (%)	100,750 (6.2)	2,150 (15.2)	1,250 (28.0)	900 (9.3)
Time variables				
Median number of days from symptoms onset to the date of patient evaluation (IQR)	3 (1-5)	2 (1-5)	4 (2-6)	2 (1-4)
Median number of days from the date of patient evaluation to death (IQR)	6 (2-12)	5 (2-11)	6 (2-12)	4 (2-10)
Median number of days from symptoms onset to death (IQR)	11 (7-17)	10 (5-16)	11 (7-18)	7 (4-14)

* Patients with missing data on predictors included in the Cox proportional-hazard regression models. Missing data were not imputed: diabetes (5,165; 0.31%), smoking habit (4,950; 0.30%), obesity (4,577; 0.28%), hypertension (4,819; 0.29%), cardiovascular disease (4,712; 0.29%), chronic obstructive pulmonary disease (4,672; 0.28%), asthma (4,644; 0.28%), chronic kidney disease (4,634; 0.28%), immunodeficiency (4,978; 0.30%), pneumonia (4,733; 0.29%), intubation (3,991; 0.24%), and admission to intensive care unit (4,281; 0.26%).

Supplementary Table 2. Characteristics of study patients without COVID-19.

Characteristic	All (n=878,840)	With diabetes (n=88,235)	Without diabetes (n=790,605)
Median age (IQR), years	40 (30-51)	56 (47-66)	38 (30-49)
Age distribution, n (%)			
20-39 years	430,225 (49.0)	9,126 (10.3)	421,099 (53.3)
40-49 years	195,850 (22.3)	17,577 (19.9)	178,273 (22.6)
50-59 years	136,403 (15.5)	25,127 (28.5)	111,276 (14.1)
60-69 years	68,230 (7.8)	20,388 (23.1)	47,842 (6.1)
70-79 years	31,843 (3.6)	11,188 (12.7)	20,655 (2.6)
≥80 years	16,289 (1.9)	4,829 (5.5)	11,460 (1.5)
Male sex, n (%)	407,961 (46.4)	41,674 (47.2)	366,287 (46.3)
Smoking habit, n (%)	85,816 (9.8)	9,069 (10.3)	76,747 (9.7)
Pneumonia, n (%)	60,618 (6.9)	18,184 (20.6)	42,434 (5.4)
Outpatient, n (%)	791,899 (90.1)	60,229 (68.3)	731,670 (92.6)
Hospitalized, n (%)	86,941 (9.9)	28,006 (31.7)	58,935 (7.5)
Admitted to intensive care unit, n (%)	5,957 (0.7)	2,021 (2.3)	3,936 (0.5)
Intubated, n (%)	8,305 (0.9)	2,886 (3.3)	5,419 (0.7)
Died, n (%)	20,134 (2.3)	7,923 (9.0)	12,211 (1.5)
Pre-existing comorbidities, n (%)			
Diabetes	88,235 (10.0)	--	--
Obesity	122,550 (13.9)	22,242 (25.2)	100,308 (12.7)
Hypertension	125,014 (14.2)	46,801 (53.0)	78,213 (9.9)
Cardiovascular disease	16,467 (1.9)	5,863 (6.6)	10,604 (1.3)
Chronic kidney disease	14,804 (1.7)	7,538 (8.5)	7,266 (0.9)
COPD	10,998 (1.3)	4,076 (4.6)	6,922 (0.9)
Asthma	26,515 (3.0)	2,938 (3.3)	23,577 (3.0)
Immunodeficiency	10,401 (1.2)	2,646 (3.0)	7,755 (1.0)
Any comorbidity including diabetes	287,000 (32.7)	--	--
Any comorbidity excluding diabetes	258,925 (29.5)	60,160 (68.2)	198,765 (25.1)
Time variables			
Person-days of follow-up	24,172,062	2,297,161	21,874,901
Median number of days from symptoms onset to the date of patient evaluation (IQR)	3 (1-5)	3 (1-5)	3 (1-5)
Median number of days from the date of patient evaluation to death (IQR)	5 (2-11)	4 (1-10)	5 (2-11)
Median number of days from symptoms onset to death (IQR)	9 (5-15)	9 (5-15)	9 (5-16)

COPD, chronic obstructive pulmonary disease.

Supplementary Table 3. Characteristics of deceased subjects.

Characteristic	With COVID-19 (n=80,616)	Without COVID-19 (n=20,134)
Median age (IQR), years	63 (54-73)	64 (53-74)
Age distribution, n (%)		
20-39 years	4,301 (5.3)	1,789 (8.9)
40-49 years	9,409 (11.7)	2,182 (10.8)
50-59 years	17,766 (22.0)	3,868 (19.2)
60-69 years	22,422 (27.8)	4,983 (24.8)
70-79 years	17,532 (21.8)	4,365 (21.7)
≥80 years	9,186 (11.4)	2,947 (14.6)
Male sex, n (%)	51,696 (64.1)	12,164 (60.4)
Smoking habit, n (%)	6,537 (8.1)	2,194 (10.9)
Pneumonia, n (%)	59,673 (74.0)	13,141 (65.3)
Outpatient, n (%)	8,749 (10.9)	1,279 (6.4)
Hospitalized, n (%)	71,867 (89.2)	18,855 (93.7)
Admitted to intensive care unit, n (%)	7,977 (9.9)	1,927 (9.6)
Intubated, n (%)	25,335 (31.4)	5,019 (24.9)
Pre-existing comorbidities, n (%)		
Diabetes	31,389 (38.9)	7,923 (39.4)
Obesity	19,840 (24.6)	3,649 (18.1)
Hypertension	36,764 (45.6)	9,204 (45.7)
Cardiovascular disease	4,347 (5.4)	1,710 (8.5)
Chronic kidney disease	5,868 (7.3)	2,514 (12.5)
COPD	3,966 (4.9)	1,577 (7.8)
Asthma	1,610 (2.0)	393 (2.0)
Immunodeficiency	1,968 (2.4)	1,110 (5.5)
Any comorbidity including diabetes	58,180 (72.2)	14,747 (73.2)
Any comorbidity excluding diabetes	51,101 (63.4)	13,244 (65.8)
Time variables		
Median number of days from symptoms onset to the date of patient evaluation (IQR)	4 (2-7)	3 (1-6)
Median number of days from the date of patient evaluation to death (IQR)	6 (3-11)	4 (1-10)
Median number of days from symptoms onset to death (IQR)	11 (7-17)	9 (5-15)

COPD, chronic obstructive pulmonary disease.

Supplementary Table 4. Association of age, sex, pre-existing clinical conditions, and smoking habit with mortality among subjects with COVID-19. *

	Hazard ratio (95% CI)	
**Variable**	**Unadjusted**	**Adjusted†**
Age group, yr		
20-39	0.11 (0.10-0.11)	0.13 (0.13-0.14)
40-49	0.42 (0.41-0.43)	0.46 (0.45-0.47)
50-59	Reference	Reference
60-69	2.17 (2.13-2.22)	1.97 (1.93-2.01)
70-79	3.51 (3.44-3.59)	3.05 (2.99-3.12)
≥80	4.42 (4.31-4.53)	4.02 (3.91-4.12)
Male sex	1.68 (1.66-1.71)	1.65 (1.63-1.68)
Diabetes	3.74 (3.69-3.80)	1.49 (1.47-1.52)
Obesity	1.49 (1.46-1.51)	1.39 (1.37-1.41)
Hypertension	3.68 (3.63-3.73)	1.23 (1.21-1.25)
Cardiovascular disease	3.13 (3.04-3.23)	0.99 (0.96-1.02)
Chronic kidney disease	4.81 (4.68-4.94)	2.01 (1.95-2.06)
Chronic obstructive pulmonary disease	3.98 (3.85-4.11)	1.21 (1.17-1.25)
Asthma	0.77 (0.73-0.81)	0.86 (0.82-0.91)
Immunodeficiency	2.55 (2.44-2.67)	1.39 (1.33-1.45)
Smoking habit	1.08 (1.05-1.11)	0.97 (0.94-0.99)

* Hazard ratios with 95% confidence intervals (CIs) were calculated using the Cox proportional-hazards regression.

† Hazard ratios were adjusted for all variables included in the model and age (a five-knot restricted cubic spline fitting was used for age), except for age group.

Supplementary Table 5. Sensitivity analysis for the association of diabetes with mortality among subjects with COVID-19.

	**Adjusted hazard ratio (95% CI) ***
Full model †	1.49 (1.47-1.52)
Full model adjusted for pneumonia	1.26 (1.25-1.28)
Full model adjusted for admission to intensive care unit	1.47 (1.45-1.49)
Full model adjusted for intubation	1.42 (1.40-1.44)
Full model adjusted for time from symptoms onset to the date of patient evaluation	1.49 (1.47-1.52)

* Hazard ratios with 95% confidence intervals (CIs) were calculated using the Cox proportional-hazards regression.

† Full model denotes the association of diabetes with mortality adjusting for age, sex, smoking habit, obesity, hypertension, cardiovascular disease, chronic obstructive pulmonary disease, asthma, chronic kidney disease, and immunodeficiency. A five-knot restricted cubic spline fitting was used for age.

Supplementary Table 6. Association of diabetes with mortality among subjects with COVID-19 according to age and sex. *

	Women		Men	
	**Number of subjects**	**Adjusted hazard ratio (95% CI)**	**Number of subjects**	**Adjusted hazard ratio (95% CI)**
**Age group, yr**				
20-39	146,521	4.09 (3.54-4.74)	150,360	2.65 (2.38-2.96)
40-49	82,251	2.91 (2.68-3.16)	87,394	2.09 (1.97-2.21)
50-59	65,587	2.06 (1.95-2.18)	73,120	1.58 (1.52-1.65)
60-69	39,173	1.53 (1.46-1.61)	47,273	1.33 (1.29-1.38)
70-79	19,749	1.26 (1.19-1.32)	25,603	1.17 (1.12-1.22)
≥80	9,097	1.12 (1.05-1.20)	11,082	1.10(1.03-1.16)

* Hazard ratios with 95% confidence intervals (CIs) were calculated using the Cox proportional-hazards regression. Estimates within each age group were adjusted for age, sex, smoking habit, obesity, hypertension, cardiovascular disease, chronic obstructive pulmonary disease, asthma, chronic kidney disease, and immunodeficiency.

Supplementary Table 7. Incidence rates of mortality among subjects with COVID-19 according to age and sex.

	Incidence rate per 100,000 person-years *					
	All		Women		Men	
	With diabetes	Without diabetes	With diabetes	Without diabetes	With diabetes	Without diabetes
**Age group, yr**						
20-39	311	44	264	26	356	62
40-49	541	160	422	92	648	227
50-59	872	392	697	242	1,041	534
60-69	1,523	942	1,290	686	1,751	1,161
70-79	2,249	1,734	1,933	1,396	2,550	1,996
≥80	2,774	2,361	2,380	1,927	3,197	2,730

* Rounded incidence rates were estimated for subjects with PCR-confirmed COVID-19 enrolled from January 1 through October 7, 2020, who were followed up for 28 days unless the event (death) occurred first.
